# A method for improved clustering and classification of microscopy images using quantitative co-localization coefficients

**DOI:** 10.1186/1756-0500-5-281

**Published:** 2012-06-08

**Authors:** Vasanth R Singan, Kenan Handzic, Kathleen M Curran, Jeremy C Simpson

**Affiliations:** 1 School of Biology and Environmental Science & Conway Institute of Biomolecular and Biomedical Research, University College Dublin, Dublin 4, Belfield, Ireland; 2 School of Medicine and Medical Science, University College Dublin, Dublin 4, Belfield, Ireland

**Keywords:** Quantitative co-localization, Image analysis, Texture features, Clustering, Rab proteins

## Abstract

**Background:**

The localization of proteins to specific subcellular structures in eukaryotic cells provides important information with respect to their function. Fluorescence microscopy approaches to determine localization distribution have proved to be an essential tool in the characterization of unknown proteins, and are now particularly pertinent as a result of the wide availability of fluorescently-tagged constructs and antibodies. However, there are currently very few image analysis options able to effectively discriminate proteins with apparently similar distributions in cells, despite this information being important for protein characterization.

**Findings:**

We have developed a novel method for combining two existing image analysis approaches, which results in highly efficient and accurate discrimination of proteins with seemingly similar distributions. We have combined image texture-based analysis with quantitative co-localization coefficients, a method that has traditionally only been used to study the spatial overlap between two populations of molecules. Here we describe and present a novel application for quantitative co-localization, as applied to the study of Rab family small GTP binding proteins localizing to the endomembrane system of cultured cells.

**Conclusions:**

We show how quantitative co-localization can be used alongside texture feature analysis, resulting in improved clustering of microscopy images. The use of co-localization as an additional clustering parameter is non-biased and highly applicable to high-throughput image data sets.

## Findings

### Background

The distribution of proteins to specific subcellular structures in eukaryotic cells allows distinct functions to be performed in parallel. Accurate determination of protein localization is therefore an essential step towards understanding cell function [1]. A variety of methods to automatically annotate subcellular localization have been described [2], primarily using supervised classification methods based on standard subcellular localization profiles [3]. One important image analysis technique for the analysis of large-scale cell-based data is texture-based analysis [4]. Of particular note are the algorithms developed by Haralick, which take account of pixel intensity information in localized areas of an image [5]. Texture-based analyses are a very powerful method to discriminate localization patterns, and as such have been implemented in various commercial and open-source image analysis solutions [6]. Despite the proven application of texture-based methods in the analysis of a variety of cell-based assays, their application in the discrimination of subtle, yet important, localization differences is less clear. For example, in eukaryotic cells proteins are rapidly being shuttled between different compartments of the endomembrane system in order to maintain secretory and endocytic pathway function. High-throughput imaging-based approaches have identified many of the molecules of these pathways [7], however automated annotation and discrimination of localization remains poor. Although clustering of proteins using texture-based features extracted from microscopy images is robust in classifying broad differences in localization, closely related proteins having similar localization profiles are not easily distinguished from one another. In this work we show how clustering using texture features can be improved with the addition of quantitative co-localization information with known organelle markers. Specifically we use a recently described algorithm, the Rank Weight Co-localization (RWC) coefficient [8], which efficiently integrates pixel co-occurrence and correlation, and demonstrate how RWC coefficients can be used as an additional feature to improve the clustering and classification of image data.

### Methods

#### Cell Culture

HeLa cells (human cervical cancer cell line, ATCC CCL-2) were routinely cultured in Dulbecco’s Modified Eagle Medium (DMEM) (Life Technologies) supplemented with 10 % foetal bovine serum (FBS) (PAA Laboratories) and 1 % L-glutamine (Life Technologies) at 37 °C in a 5 % CO_2_ incubator. Cells were sub-cultured at 1:10 dilution by incubation with 0.5 % trypsin / 0.2 % EDTA (Sigma) on reaching confluency, typically every 2 days. Cells were not used beyond passage 15.

#### cDNA Transfection & Cell Fixation

Prior to the day of transfection, 30,000 HeLa cells were plated into each well of a 12-well plate containing coverslips. On the day of transfection the cells were transiently transfected with DNA constructs encoding various fluorescently-labelled (mCherry) small GTP binding proteins of the Rab family, specifically Rab1B, Rab3C, Rab6A, Rab14, Rab33B and Rab43 using FuGENE6 (Roche). Briefly, 1.5 μl of FuGENE6 was diluted with 50 μl of OptiMEM (Life Technologies) and incubated for 5 minutes at room temperature. The diluted transfection reagent was then added to 0.5 μg of DNA and incubated at room temperature for 45 minutes. The transfection complexes were added drop-wise to the cells and incubated for a total of 24 hours. cells were fixed with 3 % paraformaldehyde (Sigma) for 20 minutes, then quenched with 30 mM glycine for 5 minutes, permeabilised with 0.1 % Triton X-100 for 5 minutes, and then washed three times with PBS. The cells were immunostained with antibodies against the *cis*-Golgi protein GM130 (BD Biosciences, cat. no. 610823) (final concentration 0.5 μg/ml), followed by anti-mouse Alexa-Fluor488 antibodies (Molecular Probes, cat. no. A11029) (final concentration 2.5 μg/ml), each for 30 minutes. The cells were then incubated in PBS containing 0.2 μg/ml Hoechst 33342 (Sigma) for 5 minutes to label the nuclei, before two final washes with PBS. The coverslips were mounted on to glass slides using Mowiol (Sigma).

#### Image Acquisition and Analysis

Confocal images (1024*1024 pixels) were acquired with an Olympus FV1000 confocal microscope equipped with a 60x/1.35 NA oil immersion objective, and using sequential scanning mode. A minimum of 40 cells were imaged for each mCherry-Rab construct tested, taken from 10 fields-of-view selected randomly. Image texture and morphological features (Haralick, Gabor, Mean Intensity and Spot-Edge-Ridge algorithms) were extracted for each individual cell using Columbus image analysis software (Perkin Elmer). Each cell was segmented using the mCherry-Rab channel to identify the cell boundary, using the ‘cytoplasm detect method B’ in the software. The software provided automated generation of texture features for each segmented cell, presented as a table containing all the texture feature results. Additional features including Radial Moments and Radial Shift Haralick features were adapted from Acapella scripts (Perkin Elmer) developed in the Andrews lab (http://www.macbiophotonics.ca/downloadsa.htm). A total of 24 features were used for this study. The RWC algorithm has been described previously [8], and for the purposes of this work was also implemented in Columbus using Acapella scripts.

#### Statistics

The ANOVA (Analysis of Variance) test was performed using R 2.12.2. The ‘aov’ routine available within the ‘stats’ package was used in conjunction with ‘DTK’ to perform a post-hoc Dunnett-Tukey-Kramer test. DTK was used for pair-wise multiple comparison tests adjusted for unequal variance and sample sizes.

#### PCA and Clustering

All analyses were performed using R 2.12.2. Principal Component Analysis (PCA) was performed using the ‘prcomp’ function within the ‘stats’ package available in R. The 24 texture features were subjected to PCA with / without RWC by scaling all the features to unit variance using the available option for scaling. The resulting principal components were clustered using the k-means clustering routine with the Hartigan-Wong algorithm, specifying 2 centres for clustering. The scores from all the principal components were used for clustering, however only the first two principal component scores (PC1 and PC2) were used for display purposes as a 2D plot, as these two components explained the majority (72 %) of the variations seen in the data.

### Results and discussion

**Figure 1:**
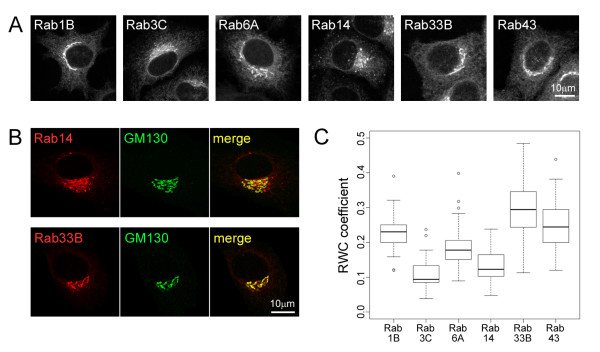
**Subcellular distribution of various Rab family small GTP binding proteins.(A)** Example images showing mCherry-Rab proteins expressed in HeLa cells. **(B)** Example dual colour images of mCherry-Rab proteins co-immunostained with the *cis*-Golgi marker GM130. **(C)** Box plot showing rank-weighted co-localization coefficients of the various mCherry-Rab proteins with GM130. The central lines within each plot represent the median co-localization values. Statistical analysis (ANOVA) revealed overall significant difference between the 6 Rabs (p < 0.001).

The endomembrane system in mammalian cells is a highly complicated set of membranes requiring large numbers of regulatory proteins to control its function. The Rab small GTPases are one such important family of regulators, having complex and overlapping membrane distributions that remain to be mapped comprehensively [9]. The distinct localization and cascade-based functioning of Rabs provides an excellent example with which to demonstrate localization-based clustering. From the 60 family members, we selected six Rabs, which have all been reported to either localize to the Golgi complex, or to other membrane compartments with a close spatial proximity to this central cellular organelle [9]. Due to their high degree of similarity, specific antibodies against all Rab family members are not available, and therefore a transfection and overexpression approach utilising Rabs tagged with the fluorescent protein mCherry was chosen. Visual examination of the expressed proteins revealed localization patterns close to the nucleus in the so-called juxta-nuclear area of the cell, which were difficult to distinguish from one another (Figure 1A). We next immunostained these cells for the protein GM130, thereby providing a reference for the *cis*-side of the Golgi complex (Figure 1B). Finally we carried out quantitative co-localization analysis using a recently described algorithm, the Rank Weight Co-localization (RWC) method [8], which uses both pixel co-occurrence and intensity correlation to generate a measure of the overlap between the two colour channels. The algorithm uses a weighting scheme to discriminate co-localizing pixels with a similar intensity from those having extreme values. The advantage of this algorithm is that co-localizing pixel positions having high intensity correlation receive higher weighting compared to positions having poor intensity correlations. This ensures that the algorithm discriminates for both co-occurrence and correlation between the two channels, and as such provides high sensitivity in co-localization analysis.

These experiments revealed a variety of RWC coefficients for the Rabs with the *cis*-Golgi marker, consistent with the notion that they all display differential localizations with respect to this organelle (Figure 1C). Statistical analysis using the ANOVA (Analysis of Variance) test revealed overall significant difference between the 6 Rabs (p < 0.001). A post-hoc Dunnett-Tukey-Kramer test revealed significant difference (p < 0.001) in mean values for all pairs of Rabs, with the exception of the Rab3C / Rab14 and the Rab1B / Rab43 pairs. Together, these experiments indicated that the RWC metric is able to differentiate some localization distribution patterns, and therefore would be a valid parameter to aid classification of localization.

**Figure 2:**
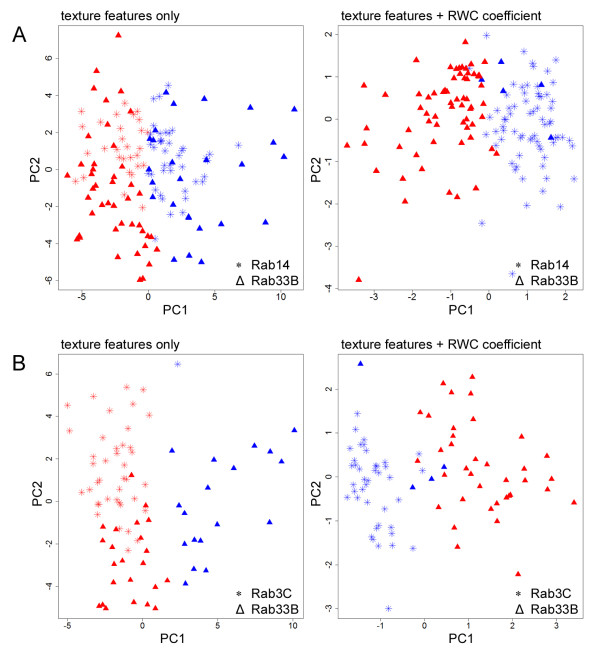
Clustering of Rab family localizations based on texture feature analysis with and without the inclusion of the co-localization metric.

We next used the images of the mCherry-Rab-expressing cells and extracted texture features for each individual cell [6]. Initially we focussed our efforts on Rab14 and Rab33B, as these two proteins have distinct functions, but yet both partially localize to the Golgi complex [10,11]. A total of 24 texture features was extracted for each cell, which were independently reduced using principal component analysis (PCA) and clustered using the k-means technique. Using standard texture analysis, a clustering accuracy of only 61 % was achieved and it was not possible to discriminate the two cell populations expressing the Rab proteins into their two correct clusters (Figure 2A, left panel). We repeated this analysis, but this time taking account of the co-localization information obtained from immunostaining with the *cis*-Golgi marker GM130 for the same set of cells (Figure 1). PCA and clustering was performed, with the principal component scores from the 24 features as independent variables and with the inclusion of the RWC co-localization coefficient as one additional feature. The principal component scores were used instead of the feature variables to avoid the problem of multi-collinearity. The new dataset with 25 features (24 texture features and 1 RWC metric) were reduced using PCA and clustered using k-means in the same manner as before. The inclusion of the RWC coefficient as an additional parameter in the analysis resulted in a clustering accuracy of 96 %, with only 5 cells from the 140 cells analysed being incorrectly classified (Figure 2A, right panel). This result was striking because these two proteins (Rab14 and Rab33B) display highly similar localization patterns (Figures 1A and 1B), which could not be separated by texture-based analysis alone. We next applied the same methodology to another Rab protein, Rab3C. This family member is believed to function in exocytosis and has been reported to localize to post-Golgi secretory vesicles [12], but again its localization pattern was indistinguishable from the other Rabs tested in this study (Figure 1A). When compared to Rab33B, texture feature analysis alone resulted in a clustering accuracy of 71 %, however with the inclusion of the RWC metric as an additional classification parameter the clustering accuracy was dramatically improved to 95 % (Figure 2B).

In this work we describe a novel use for the co-localization metric, and demonstrate how it can be applied to improve the clustering of microscopy image data. Importantly this method has the advantage that it can be applied in an unsupervised clustering format, unlike texture analysis alone that usually requires previously known groups for classification. Furthermore, as the RWC method works independently of manual intervention such as thresholding, this methodology is suitable for the analysis of large-scale image data sets coming from high content screens. Here we demonstrate how this methodology can be used to separate proteins from a single family that show highly similar localization patterns in the highly congested juxta-nuclear region of the cell. We envisage that this approach could be easily applied to a wide variety of cellular structures and organelles and thereby assist in their functional characterization.

## Availability and requirements

The RWC scripts are implemented in ImageJ (NIH) and Acapella Studio (Perkin Elmer). The Acapella version requires the Columbus Image Data Storage and Analysis System from Perkin Elmer. All scripts and representative images described in this work are available at: http://simpsonlab.pbworks.com/w/page/48541482/Bioinformatic_Tools.

## Abbreviations

PCA, Principal Component Analysis; RWC, Rank Weight Co-localization.

## Competing interests

The authors declare that they have no competing interests.

## Authors’ contributions

JCS conceived the study; JCS and VRS prepared the figures and manuscript; VRS and KH performed the experiments and carried out the analysis. All authors read, contributed to and approved the final manuscript.
